# Parental self-efficacy in childhood overweight: validation of the Lifestyle Behavior Checklist in the Netherlands

**DOI:** 10.1186/1479-5868-10-7

**Published:** 2013-01-18

**Authors:** Sanne MPL Gerards, Karin Hummel, Pieter C Dagnelie, Nanne K de Vries, Stef PJ Kremers

**Affiliations:** 1 Department of Health Promotion, Maastricht University, Maastricht, the Netherlands; 2 School for Nutrition, Toxicology and Metabolism (NUTRIM), Maastricht University, Maastricht, the Netherlands; 3 School of Public Health and Primary Care (CAPHRI), Maastricht University, Maastricht, the Netherlands; 4 Department of Epidemiology, Maastricht University, Maastricht, the Netherlands

**Keywords:** Childhood obesity, Parenting, Self-efficacy, Validation

## Abstract

**Background:**

Evaluating whether parental challenges and self-efficacy toward managing children’s lifestyle behaviors are successfully addressed by interventions requires valid instruments. The Lifestyle Behavior Checklist (LBC) has recently been developed in the Australian context. It consists of two subscales: the Problem scale, which measures parental perceptions of children’s behavioral problems related to overweight and obesity, and the Confidence scale, measuring parental self-efficacy in dealing with these problems. The aim of the current study was to systematically translate the questionnaire into Dutch and to evaluate its internal consistency, construct validity and test-retest reliability.

**Methods:**

The LBC was systematically translated by four experts at Maastricht University. In total, 392 parents of 3-to13-year-old children were invited to fill out two successive online questionnaires with a two-week interval. Of these, 273 parents responded to the first questionnaire (test, response rate = 69.6%), and of the 202 who could be invited for the second questionnaire (retest), 100 responded (response rate = 49.5%). We assessed the questionnaire’s internal consistency (Cronbach’s α), construct validity (Spearman’s Rho correlation tests, using the criterion measures: restrictiveness, nurturance, and psychological control), and test-retest reliability (Spearman’s Rho correlation tests).

**Results:**

Both scales had high internal consistency (Cronbach’s α ≥ 0.90). Spearman correlation coefficients indicated acceptable test-retest reliability for both the Problem scale (r_s_ = 0.74) and the Confidence scale (r_s_ = 0.70). The LBC Problem scale was significantly correlated to all criterion scales (nurturance, restrictiveness, psychological control) in the hypothesized direction, and the LBC Confidence scale was significantly correlated with nurturance and psychological control in the hypothesized direction, but not with restrictiveness.

**Conclusions:**

The Dutch translation of the LBC was found to be a reliable and reasonably valid questionnaire to measure parental perceptions of children’s weight-related problem behavior and the extent to which parents feel confident to manage these problems.

## Background

The prevalence of childhood overweight and obesity is steadily increasing worldwide [1]. In the Netherlands, 13-15% of the Dutch children were overweight in 2009, a two- to three-fold increase relative to 1980 figures [2]. In addition, two percent of the children were obese, which is four to six times the prevalence in 1980. In response to this increase, an increasing number of interventions have been developed with the aim of preventing or treating overweight and obesity in children. A substantial number of these interventions are aimed at parents [3-5], who are important contributors to children’s energy balance-related behaviors (i.e. food intake and physical activity behaviors that are primary determinants of weight gain [6]) and weight status. Evaluating the effects of parenting interventions on relevant intermediate outcome measures is necessary to get insight in working mechanisms of interventions. However, for this purpose, validated instruments are required.

Parenting can be a challenging job. Parents generally receive little preparation apart from having been parented themselves, so most parents learn by trial and error [7]. In identifying parenting-related behaviors, two levels are often distinguished [8]: specific parenting practices and general parenting styles. Specific parenting practices are behaviors that relate to a specific domain (e.g. nutrition or physical activity). Examples of specific parenting practices are rules about having breakfast or controlling the availability of fruit at home. General parenting styles reflect the emotional climate in which behavior-specific parenting takes place, which determines the context in which parent–child interactions occur [9]. Examples of parenting styles include responsiveness (extend to which parents are aware of their children’s feelings, problems and difficulties and the way they respond in a supportive way), or demandingness (controlling children’s behaviors). In the nutrition and parenting literature, both levels of parenting have been shown to be of importance in explaining and predicting children’s energy balance-related behaviors [8]. In addition, parenting behaviors that may contribute to a child’s positive energy balance seem to be positively associated with parental weight (or BMI) [10,11].

In contrast to parenting practices and styles, the construct of weight-related parenting self-efficacy has been largely neglected in the research on food and activity parenting [12,13]. According to Bandura [14], self-efficacy is ‘a person’s belief in his capabilities to organize and execute the course of action required to manage prospective situations’. In the general parenting literature, self-efficacy is recognized as an important determinant of parenting behaviours [15,16]. As acknowledged by the few prior studies in this area [12,13,17], low parental self-efficacy may be a barrier for parents trying to change their children’s nutrition and physical activity behaviors. Compared to parents with healthy children, parents of overweight and obese children may face additional challenges in the upbringing of their children, including being worried about stigmatization of their child, wanting to protect their child from stigmatization, feeling ambivalent about setting limits for their child, uncertainty about being a good parent, and uncertainty about, on the one hand, accepting the child as he/she is and, on the other hand, feeling responsible for their child’s health [18]. Mothers of overweight and obese children have reported frustration as a result of their children’s unwillingness to eat a healthy diet and be physically active [19]. This overweight-specific parental self-efficacy differs in nature from behavioral practices and styles, but it seems to be an essential additional component for parents to succeed in raising their children to become healthy adults.

As a first start towards developing a parenting intervention, it is valuable to identify initial parental challenges in managing children’s lifestyle behavior and to assess parental self-efficacy. It is also useful to investigate whether parental confidence and skills regarding weight-related challenges are addressed by interventions. In view of the lack of a specific instrument to measure weight-related parental self-efficacy, West and colleagues [20] developed the Lifestyle Behavior Checklist (LBC), a tool to measure parental perceptions of their children’s behavioral problems with overweight and obesity, and parents’ self-efficacy in dealing with these behaviors.

West and colleagues performed two studies in Australia to test the validity of the LBC [17,21]. They tested its content validity by determining whether the LBC could be used to distinguish between families with and families without obese children. Parents of children with a healthy weight reported lower levels of lifestyle behavior problems and higher levels of parental self-efficacy. Furthermore, the construct validity of the LBC questionnaire was assessed by using general parenting measures as criterion measures; both scales were significantly correlated with the measures of general parenting, indicating that general parenting skills are to some extent reflected in parental self-efficacy in childhood overweight. In addition, evidence suggests that the LBC scales are responsive to change following a parenting intervention [22].

The aim of the current study was to test psychometric properties of the LBC questionnaire in the Dutch context. We translated the LBC questionnaire from English into Dutch. We also tested the construct validity of the Dutch version of the LBC using general parenting style measures as criterion variables. Finally, we determined the test-retest reliability of the Dutch version of the questionnaire, by sending participants the questionnaire twice with a two-week interval.

## Methods

### Overview of procedures and participants

**Figure 1:**
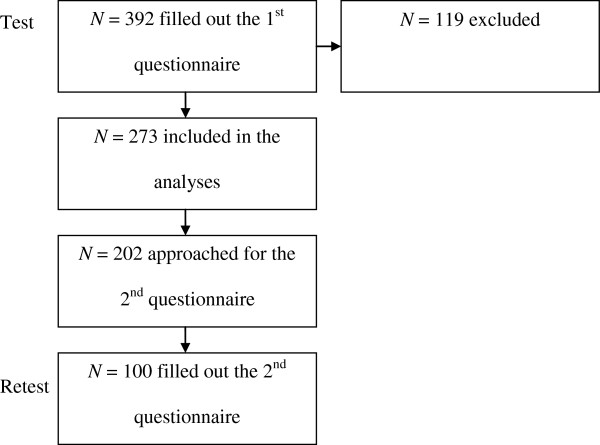
Flow diagram of participants.

Potential participants were invited to participate via an online survey panel (Thesistools, The Hague). This panel consists of participants who receive an invitation to participate in a survey once a month. They do not receive a reward for participation. Respondents were included if they were parents of children aged 3–13 years, and living in the Netherlands. Two weeks after they filled out the first questionnaire, a second questionnaire (retest; LBC only) was sent to respondents who had provided their email addresses. Of the 392 participants who were exposed to the questionnaire as intended, 273 were included in the analyses. Reasons for exclusion were: not being a parent of a 3-to 13-year-old child (N = 33), more than 10% of the answers missing (N = 45), or being a parent of an underweight child (<5^th^ percentile) (N = 41). Of the 273 participants included in the analysis, 202 (74.0%) gave their permission to be invited for the second questionnaire by email. Of these, 100 responded to the second questionnaire (response rate for second questionnaire = 49.5%). These numbers are depicted in the flowchart in Figure 1.

### Measures

#### The Lifestyle Behavior Checklist

The LBC is a 25-item questionnaire which assesses parental perceptions of the extent of behavior problems of overweight and obese children and parental confidence about managing these problems [17]. The questionnaire assesses a list of 25 child problem behaviors related to eating (e.g., eats too much, argues about food), activity (e.g., watches too much television, refuses to do physical activity), and overweight (e.g., complains about being overweight, complains about not fitting into clothes). The questionnaire consists of a Problem scale and a Confidence scale. The Problem scale measures the extent to which parents perceive each of the 25 behaviors as a problem for them with their child, on a 7-point scale from 1 (not at all) to 7 (very much). The Confidence scale measures the extent to which parents feel confident about managing each of the behaviors, on a 10-point scale from 1 (certain I cannot do it) to 10 (certain I can do it). The scores on the Problem scale and the Confidence scale are combined into two sum scores, ranging from 26 to 182 and from 26 to 260, respectively.

#### Translation procedure

The LBC was translated into Dutch by four experts at Maastricht University (the Netherlands) who are also authors of this manuscript (SMPLG, KH, PCD, SPJK). The translation procedure was as follows. First, all translators independently translated the questionnaire. Inconsistencies were then discussed in a plenary session until consensus was reached and a provisional version of the questionnaire was developed. This provisional questionnaire was pretested among 2 students and 3 parents who were part of the target population. The pretest was based on cognitive interviewing, i.e. using verbal probing techniques [23]. Subsequently, another meeting between the experts took place to discuss the results of the pretest. In case of uncertainties, we contacted the developer of the questionnaire (F. West) [20]. All translators approved the final translation.

#### Demographics

Participants were asked to specify their relation to the child (biological mother, biological father, stepmother, stepfather, or other), their educational level, and the educational level of their partner, their employment status, and the employment status of their partner. Educational level was categorized into three different levels, i.e. low (primary school or lower secondary vocational education), medium (junior general secondary education, senior secondary vocational education, senior general secondary education or pre-university education), and high (higher professional education or university education). Employment status was divided into two categories, viz. employed or not.

Additional questions concerned the date of birth, height and weight of both parents. Items regarding the child included date of birth (to determine age), height and weight, gender and number of siblings. Height and weight of the parents and children were used to calculate BMI (weight (kg) / height (m))^2^. Children’s BMI was recoded into BMI z-scores compared to the 1997 national reference population (Fourth Dutch National Growth Study) [24]. Weight status was classified into healthy weight (5^th^-84^th^ percentile), overweight (85^th^–94^th^ percentile) or obesity (≥95^th^ percentile) [25].

#### General parenting

Items from the Child Rearing Practices Report scale as well as items from the Psychological Control scale, both validated Dutch versions, were included in the questionnaire as criterion validation scales to assess three theory-based general parenting dimensions: restrictiveness, nurturance and psychological control [26,27]. The Child Rearing Practices Report (CRPR) in its original form consists of 91 items to assess parents’ child-rearing attitudes, values, behaviors and goals [28]. The CRPR has been shown to be a valid instrument for assessing child-rearing dimensions in the Dutch population [29]. In the current study, we included 35 CRPR items, based on validation studies of shorter versions of the questionnaire [29,30]. The CRPR items can be divided into two scales: a restrictiveness scale and a nurturance scale. Restrictiveness items (n = 18, Cronbach’s α = 0.80) are characterized by a high degree of control, narrow limit setting, and endorsing of strict rules, requirements and restrictions (e.g. ‘I try to keep my children away from children or families whose ideas or values are different from our own’). The items on the nurturance scale (n = 17, Cronbach’s α = 0.78) reflect parents’ willingness to listen to their children and share feelings and experiences with them, parents’ responsiveness to their children’s needs, and the extent to which parents show affection and acceptance (e.g. ‘I respect my child’s opinion and encourage him to express it’).

In addition, eight psychological control items (Cronbach’s α = 0.72) from the Psychological Control Scale [27] were added in the questionnaire to include a relevant third parenting dimension [27]. Psychological control is defined as ‘parental behaviors (such as guilt induction, love withdrawal or contingent love, instilling anxiety, and invalidation of the child’s perspective) that are intrusive and manipulative to children’s thoughts, feelings, and attachments to parents’ [27]. An example item is ‘I am less friendly with my child when he/she does not see things my way’.

### Data analysis

SPSS 17.0 was used for the analyses. Descriptive statistics were used to calculate means and standard deviations of the quantitative continuous variables, and to calculate percentages of the categorical data. To determine the internal consistency of the scales, we calculated Cronbach’s α. Spearman’s Rho correlation tests were used to determine test-retest reliability.

Group means (healthy weight vs overweight children) on the individual LBC items and the LBC scale scores were compared using one-way ANOVA. We applied ANOVA tests for the LBC scales scores with corrections for parenting constructs and demographic variables (educational status of both parents, employment status of both parents, maternal and paternal age, age of the child, BMI of both parents and BMI z-score of the child).

Construct validity between the LBC scales and the parenting styles scales was assessed using bivariate correlations (Spearman’s Rho correlation tests) and partial correlations, corrected for the demographics. We used the magnitude of the relationship (‘effect size’) (r and partial r) as a source of information. Interpretation of the strength of the effect size was based on Cohen’s descriptive guidelines [31]. A correlation higher than or equal to 0.50 was regarded as a large effect size, correlations between 0.30 and 0.50 as medium effect size, and a correlation higher than or equal to 0.10 as a small effect size. With regard to the partial r, a small effect size was defined as one larger than or equal to 0.02, a medium effect as larger than 0.15, and a large effect size as larger than or equal to 0.35.

## Results

### Characteristics of the participants

Characteristics of the test and retest population are summarized in Table 1. In most cases, it was the biological mother of the child who filled out the questionnaire. Most parents had a high educational level (64.8% of mothers and 59.7% of fathers) and were employed (79.1% of mothers and 90.5% of fathers). About one third of the mothers were overweight, and about 11% were obese. Half of the fathers were overweight or obese. Regarding the weight status of the children, 11.4% of the test sample was overweight or obese, compared to 13.0% of the retest sample. We tested whether we could predict drop-out with respect to the test-retest samples using demographics. No statistically significant predictors were found, indicating non-selective drop-out.

**Table 1:** Characteristics of the samples

**Variable**	**Test sample(*N* = 273)**		**Retest sample(*N*** = **100)**	
**Continuous**	**Mean(sd)**		**Mean(sd)**	
Age (years)				
*Mother*	40.35 (7.01)		41.70 (6.75)	
*Father*	42.84 (7.33)		44.20 (7.25)	
*Child*	7.88 (2.73)		8.08 (2.78)	
Number of siblings	2.30 (1.04)		2.25 (1.03)	
BMI				
*Mother*	24.93 (4.18)		25.12 (4.34)	
*Father*	25.33 (3.34)		25.25 (3.43)	
*Child*	16.31 (2.24)		16.51 (2.18)	
BMI z-score child	−0.08 (1.00)		−0.03 (0.94)	
**Categorical**	**N**	**%**	**N**	**%**
Child sex				
*Male*	140	51.3	50	50.0
*Female*	133	48.7	50	50.0
Relation to child				
*Biological mother*	209	76.6	78	78.0
*Biological father*	56	20.5	19	19.0
*Stepmother*	2	0.7	1	1.0
*Stepfather*	0	0	0	0
*Other*	6	2.2	2	2.0
Mother’s education				
*Low*	4	1.5	1	1.0
*Medium*	86	31.5	27	27.0
*High*	177	64.8	70	70.0
*Missing*	6	2.2	2	2.0
Father’s education				
*Low*	16	5.9	5	5.0
*Medium*	88	32.2	27	27.0
*High*	163	59.7	66	66.0
*Missing*	6	2.2	2	2.0
Mother employed				
*No*	51	18.7	18	18.0
*Yes*	216	79.1	80	80.0
*Missing*	6	2.2	2	2.0
Father employed				
*No*	20	7.3	9	9.0
*Yes*	247	90.5	89	89.0
*Missing*	6	2.2	2	2.0
Mother’s weight category				
*Underweight*	3	1.1	1	1.0
*Healthy weight*	150	54.9	57	57.0
*Overweight*	90	33.0	31	31.0
*Obesity*	30	11.0	11	11.0
Father’s weight category				
*Underweight*	0	0	0	0
*Healthy weight*	139	50.9	47	47.0
*Overweight*	110	40.3	47	47.0
*Obesity*	24	8.8	6	6.0
Child’s weight category				
*Healthy weight*	242	88.6	87	87.0
*Overweight*	16	5.9	6	6.0
*Obesity*	15	5.5	7	7.0

### Reliability

Means and standard deviations of the Problem and Confidence scales are listed in Table 2. Both the Problem scale (Cronbach’s α test = 0.92, retest = 0.91) and the Confidence scale (Cronbach’s α test = 0.98, retest = 0.90) had high internal consistency. Spearman correlation coefficients, to determine test-retest reliability, were acceptable for the Problem scale (r_s_ = 0.74, p < 0.001), as well as the Confidence scale (r_s_ = 0.70, p < 0.001).

**Table 2:** Mean scores of study sample on parenting scales

	**Range**	**Test sample**	**Retest sample**	
		**(*N* = 273)**	**(*N* = 100)**	
		**Mean(sd)**	**Mean(sd)**	
			**Test scores**	**Retest scores**
LBC Problem scale	26-182	39.12 (14.00)	38.15 (10.27)	39.18 (12.63)
LBC Confidence scale	26-260	208.14 (32.85)	210.37 (28.63)	205.57 (28.78)
Nurturance	1-5	4.51 (0.33)	4.53 (0.28)	-
Restrictiveness	1-5	2.48 (0.47)	2.42 (0.47)	-
Psychological control	1-5	1.79 (0.53)	1.72 (0.50)	-

### Group differences

The scores on individual items of the Problem scale and the Confidence scale were compared between parents of healthy weight and overweight (including obese) children (see Table 3). With regard to the Problem scale items, parents of overweight children scored significantly higher on 14 of the 25 items compared to parents of healthy weight children. On four confidence items, parents of overweight children scored significantly lower than parents of normal weight children. Other Confidence scale items did not significantly differ between the parents of non-overweight and those of overweight children.

**Table 3:** Group means for the LBC items

	**Problem scale**						**Confidence scale**					
	**Healthy weight**		**Overweight + obese**				**Healthy weight**		**Overweight + obese**			
	**(*N* = 242)**		**(*N*** = **31)**				**(*N* = 242)**		**(*N* = 31)**			
**Item *My child***…	**Mean**	**sd**	**Mean**	**sd**	**F**	**p**	**Mean**	**sd**	**Mean**	**sd**	**F**	**p**
1. Eats too quickly	1.24	0.65	1.81	1.33	15.11	<0.001	8.72	1.42	8.29	1.70	2.40	0.123
2. Eats too much food	1.33	0.78	2.45	1.59	42.30	<0.001	8.58	1.51	8.03	1.70	3.48	0.063
3. Eats unhealthy snacks	1.93	0.95	2.74	1.55	17.20	<0.001	8.34	1.48	7.48	1.75	8.78	0.003
4. Whinges or whines about food	2.45	1.24	2.84	1.37	2.57	0.110	8.15	1.43	7.74	1.79	2.09	0.150
5. Yells about food	1.28	0.81	1.29	0.90	0.00	0.952	8.85	1.10	9.01	0.95	0.64	0.425
6. Throws a tantrum about food	1.24	0.76	1.45	1.23	1.88	0.172	8.44	1.75	8.35	2.04	0.06	0.808
7. Refuses to eat certain foods (i.e. fussy eating)	2.62	1.53	2.63	1.70	0.00	0.966	7.98	1.78	7.81	1.70	0.28	0.600
8. Argues about food (e.g. when you say *No more*)	1.54	0.89	1.81	0.95	2.35	0.126	8.44	1.54	8.06	1.48	1.67	0.197
9. Demands extra helpings at meals	1.19	0.61	1.61	0.84	12.04	0.001	8.68	1.46	8.48	1.41	0.51	0.476
10. Requests food continuously between meals	1.63	0.93	2.52	1.63	20.41	<0.001	8.49	1.40	8.13	1.50	1.82	0.178
11. Demands food when shopping or on outings	1.55	0.86	1.77	1.02	1.81	0.180	8.63	1.42	8.55	1.23	0.10	0.754
12. Sneaks food when they know they are not supposed to	1.35	0.79	1.84	1.53	8.00	0.005	8.38	1.67	7.84	1.99	2.73	0.100
13. Hides food	1.16	0.57	1.35	1.08	2.56	0.111	8.43	1.71	7.97	1.87	1.94	0.165
14. Steals food (e.g. from other children’s lunchboxes)	1.12	0.67	1.26	1.13	1.02	0.315	8.44	1.81	8.23	1.65	0.39	0.535
15. Eats food to comfort themselves when feeling let down or depressed	1.19	0.74	1.74	1.37	12.08	0.001	8.36	1.81	8.03	1.85	0.87	0.351
16. Watches too much television	2.36	1.14	3.19	1.60	13.43	<0.001	8.03	1.48	7.39	1.65	5.09	0.025
17. Spends too much time playing video or computer games	1.90	1.16	2.48	1.36	6.62	0.011	8.23	1.40	7.90	1.51	1.46	0.228
18. Complains about doing physical activity (e.g. *This is boring*, *I*’*m too tired*, *My leg hurts*)	1.71	1.07	2.48	1.69	12.43	<0.001	8.22	1.64	7.52	2.03	4.78	0.030
19. Refuses to do physical activity	1.32	0.88	2.26	1.79	23.22	<0.001	8.38	1.65	7.74	2.16	3.76	0.054
20. Complains about being unfit or feeling low in energy	1.21	0.71	1.58	0.99	6.68	0.010	8.53	1.58	8.00	1.63	3.04	0.082
21. Complains about being overweight	1.24	0.84	1.97	1.58	16.19	<0.001	8.42	1.71	7.74	1.95	4.23	0.041
22. Complains about being teased	1.57	1.07	1.74	1.09	0.79	0.376	7.85	1.87	7.84	2.08	0.00	0.981
23. Complains about not having enough friends	1.33	0.82	1.55	1.00	1.80	0.181	7.90	1.86	8.03	1.91	0.15	0.704
24. Complains about being unattractive	1.21	0.69	1.29	0.74	0.38	0.539	8.14	1.79	8.26	1.55	0.11	0.737
25. Complains about not fitting into clothes	1.17	0.65	1.55	1.06	7.76	0.006	8.50	1.65	8.23	1.54	0.78	0.378

The ANOVA analyses of the LBC scale scores revealed a significant difference in scores onto the Problem scale between children with and without overweight (F (1, 246) = 16.94, p < 0.001). Parents in the healthy weight group scored significantly lower on the Problem scale (M = 37.83, SD = 13.27), compared to those in the overweight group (M = 49.21, SD = 15.57). The group effect on the Problem scale remained significant after correcting for the covariates (parenting constructs and demographic variables), (F (1, 237) = 11.48, p = 0.001). There was no group effect for the Confidence scale (F (1, 246) = 1.47, p = 0.227): the parents of healthy weight children (M = 209.06, SD = 33.01) did not score significantly higher on the Confidence scale than those of the overweight children (M = 200.66, SD = 31.07). The effect of the group on the Confidence scale, corrected for covariates (parenting constructs and demographic variables), was somewhat higher than the uncorrected effect (viz. F (1, 237) = 1.49, p = 0.224), but still not statistically significant.

### Construct validity

The LBC scales were tested for construct validity using the parenting style dimensions and weight measures of both parents and children. Results are shown in Table 4. We found significant correlations (unadjusted) between the LBC Problem scale and all criterion scales. The Problem scale was negatively correlated to nurturance (positive parenting dimension), and positively correlated to restrictiveness (negative parenting dimension), psychological control (negative parenting dimension) and the weight status of the child and both parents. The effect sizes of these correlations were small. After correction for demographics and parenting constructs, the Problem scale was significantly correlated to nurturance, BMI z-score of the child, and BMI scores of both parents.

**Table 4:** Correlation coefficients between the LBC scales and the criterion measures

***LBC scale***	**LBC Problem scale**		**LBC Confidence scale**	
***Criterion scales***	**Unadjusted (r**_**s**_**)**	**Adjusted for parenting and demographics**^**Â§**^	**Unadjusted (r**_**s**_**)**	**Adjusted for parenting and demographics**^**Â§**^
Nurturance	−0.23**	−0.20**	0.14*	0.04
Restrictiveness	0.14*	0.06	0.04	0.13*
Psychological control	0.19**	0.05	−0.22**	−0.18**
BMI z-score of child	0.21**	0.21**	−0.02	−0.06
BMI of mother	0.23**	0.18**	−0.06	−0.06
BMI of father	0.14*	0.16**	−0.02	−0.03

Note *p < 0.05, ** p < 0.01; r ≥ 0.10 small effect size, r ≥ 0.30 medium effect size, r ≥ 0.50 large effect size; partial r ≥ 0.20 small effect size,

partial r ≥ 0.15 medium effect size, partial r ≥ 0.35 large effect size.

^Â§^ Adjusted for the other two parenting dimensions and demographics including educational status of both parents, employment status of both parents, maternal and paternal age, age of the child, BMI z-score of the child, and BMI of the father and the mother.

Furthermore, we found an interaction effect of child weight status and the LBC Problem scale on nurturance. This means that the correlation between nurturance and the LBC Problem scale was different for parents of healthy weight children (unadjusted r_s_ = −0.20; adjusted r = −0.14) than for parents overweight and obese children (unadjusted r_s_ = −0.54; adjusted r = −0.48).

The LBC Confidence scale was positively correlated to nurturance and negatively correlated to psychological control. Again, both effect sizes were small. The Confidence scale did not correlate with the other criterion measures. The adjusted scores of the Confidence scale were positively correlated to restrictiveness and negatively correlated with psychological control. All adjusted effect sizes were small.

## Discussion

There is a need for instruments to assess parents’ problems regarding their children’s overweight and parents’ self-efficacy in managing these problems. The Lifestyle Behavior Checklist (LBC) could be a valuable addition to existing parenting instruments, especially if it can be shown to have good psychometric properties. The present study was the first to validate the LBC outside Australia (cross-national validation). In the Dutch context, the translated LBC was found to be a reliable and reasonably valid questionnaire to measure weight-related parental self-efficacy. However, the questionnaire appeared to be somewhat less valid in our sample than in the Australian validation studies.

The LBC Problem scale was significantly negatively correlated to the general parenting construct nurturance (positive parenting dimension), and positively correlated to restrictiveness (negative parenting dimension) and psychological control (negative parenting dimension). The Confidence scale was negatively correlated to psychological control, and positively correlated to nurturance. These small but significant correlations indicated that the parenting constructs were related but not identical to the Problem and Confidence scales. The correlations were in the hypothesized direction. In the Australian validation study [17], the LBC Confidence scale was found to correlate moderately well with the Parenting Scale by Arnold [32], which measures ineffective parenting (including permissive or authoritarian discipline).

Internal consistency of both scales was relatively high in both the test and retest. Correlation coefficients indicated relatively high test-retest reliability. These were comparable to the scores reported by the Australian validation study (r_s_ = 0.87 for the Problem scale, r_s_ = 0.66 for the Confidence scale).

The Confidence scale seemed to be less sensitive than the Problem scale as regards detecting differences between parents of healthy weight children and parents of overweight children. In the Australian validation study [17], statistically significant differences between groups with different weight status were found for both scales. However, that study did not compare parents of overweight children with those of healthy weight children, but compared parents of healthy weight children with those of obese children. This difference in samples probably explains why the mean scores of parents of Australian obese children on all Problem scale items were substantially higher than the scores of the parents of overweight children in our sample. Scores on Confidence scale items were substantially lower among parents of obese children in the Australian study.

We found an interaction effect between child weight status and the Problem scale for nurturance. For parents of overweight and obese children, there was a high negative correlation between the LBC Problem scale and nurturance, whereas a small negative correlation between the LBC Problem scale and nurturance was found for the parents of healthy weight children. We already knew from an earlier review [8] that the parenting dimension nurturance was positively related to overweight-preventing behaviors. Parents of overweight children may have a different parenting style than those of healthy weight children. The finding in the current study that nurturance by parents of overweight children is strongly negatively correlated to children’s weight-related problem behaviors confirms the protecting influence of nurturance.

The LBC includes 15 items related to dietary behavior, while only 4 items are related to physical activity or sedentary behavior and 6 items are related to the child’s overweight. Although an increasing number of studies have shown the importance of sedentary behavior in determining the development of overweight and obesity [33], it is conceivable that the relatively high proportion of diet-related items is in line with the actual everyday concerns of parents. Parents may indeed have more concerns about feeding their child [34], whereas they may not have too many concerns about their child watching too much television [35] or not being physically active. Earlier studies even found that parents often do not know that watching too much television is related to the development of obesity [35,36].

The LBC may also serve as a basis for an intervention or recruitment. It can be an important instrument to map parental problems, as it may also provide us with an opportunity to make parents aware of possible problems regarding to their children’s overweight. We know that the programs aimed at the prevention of obesity often struggle with recruitment problems [37,38]. However, when parents themselves recognize their child’s overweight problems, they may be more willing to take action and participate in prevention programs.

Some strong and weak points of the current study should be acknowledged. A strong point of the current study was the quality of the translation process. Four experts independently translated the questionnaire and a qualitative pretest was used to optimize the translation. However, we did not back-translate the questionnaire, which could have had additional value to the translation process. We also evaluated test-retest reliability of the LBC questionnaire. Another strong point was the relatively large sample we were able to include in the current study, making it more likely that the results can be generalized to a larger population. We recruited participants via an internet-based survey, which is known for its access to hidden populations [39]. Nevertheless, several groups of people were underrepresented compared to the general Dutch population, as parents with a low educational level, and parents of children with overweight and obesity were somewhat underrepresented. The present study lacked a test of other types of validity (e.g., discriminant validity), implying that we only partly showed evidence for the construct validity of the scale. Please note evidence for discriminant validity of the LBC has been provided in a previous study [17]. It should also be noted that the response on the retest was relatively low, limiting external validity of the study. However, we tested whether drop-out was selective, which was not the case. Furthermore, weight and height measures were self-reported which may be a reason for the apparent lower child’s weight status in our sample, compared to the Dutch population [2].

We recommend that experts who develop and evaluate interventions to prevent and treat childhood obesity should also make use of measures of parents’ self-efficacy in managing their child’s energy balance-related behaviors, to assess changes in parental perceptions of their child’s weight-related problems. The LBC can be a reliable and valid instrument to assess these intermediate intervention outcomes.

## Conclusions

The Dutch translation of the Lifestyle Behavior Checklist seems to be a reliable and, reasonably valid questionnaire to measure parents’ perception of their children’s weight-related problem behavior and the extent to which parents feel confident about managing these problems.

## Abbreviations

BMI: Body Mass Index; CRPR: Child Rearing Practices Report; LBC: Lifestyle Behavior Checklist.

## Competing interests

The authors declare that they have no competing interests.

## Authors’ contributions

SMPLG, KH, PCD and SPJK were involved in the translation procedure of the questionnaire. SMPLG and KH collected the data. SMPLG analyzed and interpreted the data, and wrote draft versions of the manuscript. KH and SPJK contributed to the interpretation of the data. KH, PCD, NKdV and SPJK were involved in revising the manuscript. All authors have read and approved the final manuscript.
